# Evaluating the Utility of Topical Haemostatic Adjuncts in Transoral Robotic Surgery (TORS)

**DOI:** 10.7759/cureus.86750

**Published:** 2025-06-25

**Authors:** Zakariya Sattar, Emma Watts, Syed Farhan Ahsan

**Affiliations:** 1 Otolaryngology - Head and Neck Surgery, New Cross Hospital, The Royal Wolverhampton NHS Trust, Wolverhampton, GBR; 2 Otolaryngology - Head and Neck Surgery, University Hospitals Birmingham, Birmingham, GBR

**Keywords:** head and neck cancer surgery, oncological head and neck surgery, topical thrombin, transoral robotic surgery, transoral robotic surgery (tors)

## Abstract

Introduction: Transoral robotic surgery (TORS) is a minimally invasive modern surgical technique, offering novel access to the oral cavity with important implications for the management of oropharyngeal squamous cell carcinoma. However, TORS carries complications including haemorrhage, pain and impaired swallowing, with potentially significant implications for patients and healthcare systems.

Aim: This combined retrospective cohort study and literature review aims to explore our experience with the use of topical haemostatic products in TORS to improve post-operative outcomes.

Methods: We analysed our experience with the use of three different topical haemostatic adjuncts during TORS procedures at a major head and neck cancer centre from October 2023 to August 2024. The primary outcome measure was the rate of post-operative haemorrhage. This cohort study was complemented by a systematic review of the existing literature regarding the use of topical haemostatic products in TORS.

Results: Eighteen patients met criteria for inclusion. The type of topical haemostatic adjunct used varied between Tisseel (n=8, 44.4%), Floseal (n=4, 22.2%) and Purabond (n=6, 33.3%). One patient (5.6%) developed a secondary post-operative haemorrhage. Another patient (n=1, 5.6%) required admission for management of post-operative pain. Our experience provides preliminary insight into the utility of topical haemostatic adjuncts in TORS and underlines the need for further research in this area.

Conclusion: Topical haemostatic adjuncts, in our view, are useful, surgeon-friendly products that may augment haemostasis in TORS. Further studies are required to determine the efficacy of individual topical haemostatic products in preventing post-operative haemorrhage and enhancing recovery following TORS.

## Introduction

Transoral robotic surgery (TORS) has revolutionised the management of oropharyngeal squamous cell carcinoma (OPSCC), allowing three-dimensional views of the surgical field, enhancing surgical precision and improving access to anatomically challenging areas of the head and neck [1]. Historically, oropharyngeal cancers or cancers of unknown primary (CUP) mandated major surgical procedures via an external approach, which is associated with significant morbidity [2]. More recently, treatment has predominantly relied on chemoradiotherapy (CRT), which also carries significant morbidity, notably in the form of mucositis and long-term dysphagia [3]. TORS was first introduced in 2005 in the USA and gained FDA approval four years later for its use in head and neck surgery [4]. This novel intervention formed a significant milestone in the management of OPSCC, leading to faster post-operative recovery, reduced pain and improved functional outcomes, reducing the requirement for adjuvant CRT [5].

A multi-centre review of over 400 patients with OPSCC by de Almeida et al. demonstrated that three-year overall survival and disease-specific survival were comparable between patients undergoing TORS with or without post-operative adjuvant chemoradiotherapy, highlighting the potential for treatment de-escalation [6]. The subsequent ORATOR clinical trial demonstrated similar side effect profiles between TORS and chemoradiotherapy, suggesting that both modalities were viable treatment options [7]. However, 70% of the TORS study population also received CRT due to their advanced nodal status, which may have confounded results. The ongoing PATHOS randomised controlled trial aims to determine whether de-escalating the intensity of adjuvant treatment for HPV-positive OPSCC improves post-operative swallowing outcomes without impacting cure rates [8].

TORS is not without post-operative complications, with rates of post-operative haemorrhage ranging from 5.4 to 9.8% [9,10]. Topical haemostatic adjuncts have been proposed as an intra-operative adjunct to reduce the risk of haemorrhage. Synthetic peptides such as Purabond form a biological matrix which encourages haemostasis [11,12]. Other products employ alternative strategies for encouraging haemostasis. Tisseel relies on a fibrin-based matrix, whilst Floseal uses a combination of gelatin and thrombin to promote coagulation [13]. These products have been proven to reduce post-operative haemorrhage across multiple surgical specialities, including ENT [14,15].

This combined technical article and systematic review focuses on our surgical experience with the use of a number of topical haemostatic adjuncts in TORS, highlighting peri-operative use of these adjuncts and the decision making concerning their application in TORS. We hope that this provides preliminary experience from which further studies can be based in order to glean further insight into the use of these products in TORS.

## Materials and methods

Cohort study

This retrospective cohort study included all patients receiving haemostatic adjuncts (Tisseel, Purabond or Floseal) during TORS at a major head and neck centre within the UK from October 2023 to August 2024. Patients were included if they underwent TORS for any indication in our regional centre. Cases that were planned to be completed robotically but were converted to conventional approaches were therefore excluded. All surgical cases were performed by a single consultant head and neck surgeon. Patient case notes were retrospectively reviewed for up to 30 days post-operatively. Data was collected regarding patient demographics, histopathological diagnosis and tumour stage. The primary outcome measure was the rate of post-operative haemorrhage, whilst length of stay, re-admission rates and swallowing outcomes were recorded as secondary outcome measures. Ethical approval was waived in view of this being a retrospective observational study in line with the Declaration of Helsinki.

Surgical technique

**Figure 1:**
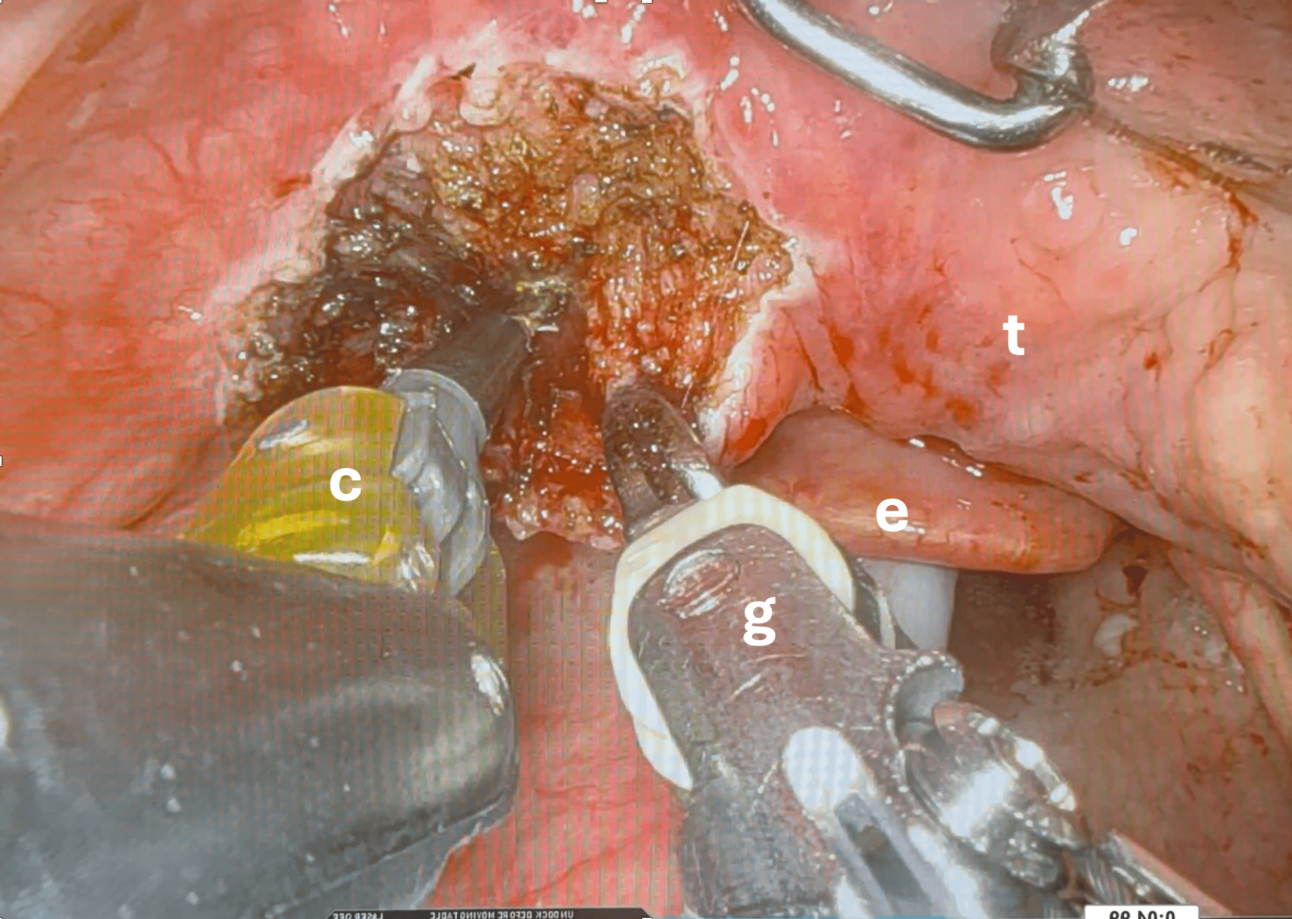
Intra-operative view of tongue base resection g: grasping forceps; c: cautery spatula; e: epiglottis; t: tongue base

**Figure 2:**
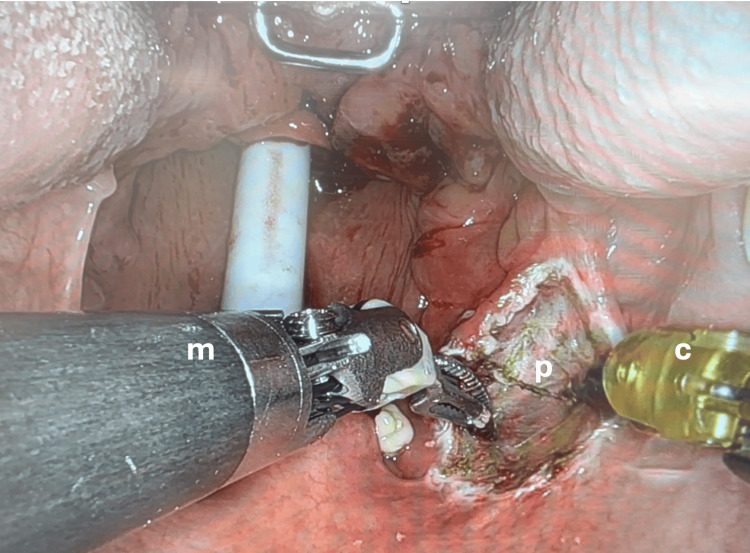
Intra-operative view at lateral oropharyngectomy m: Maryland bipolar forceps; c: cautery spatula; p: pharyngeal wall

The daVinci Xi robotic surgical system (Intuitive, US) was used in all patients in this study. A Boyle-Davis mouth gag is used to retract the tongue and mandible inferiorly. After calibration and docking of the robot, various EndoWrist instruments are deployed in order to facilitate resection of the anatomical area in question (Figures 1, 2). Preferred instruments in this series include the Maryland bipolar forceps, grasping forceps and cautery spatula (Intuitive, US). These instruments are used to initially demarcate the area of intended resection by superficially burning mucosa, then performing controlled resection under direct vision to ensure adequate depth of resection. The first assistant, positioned at the patient, can also be utilised to perform suction, retraction or aid in specimen retrieval.

Following resection and adequate haemostasis, one of the abovementioned adjuncts was prepared according to the manufacturer's instructions and applied intra-operatively to the area of resection. Application of the desired haemostat in question is usually performed with a long-tipped applicator, enabling unobstructed access to the resection site. Nasogastric tube insertion was performed prophylactically intra-operatively in anticipation of reduced oral intake. Following TORS, neck dissection or external carotid artery ligation can be performed as necessary based on prior clinical assessment.

Rationale for the selection of the haemostatic adjuncts in question depends on a number of factors. Tisseel requires thawing prior to use of up to 90 minutes depending on the method employed [16], whilst Floseal can be prepared in as little as 30 seconds [17]. Purabond does not require any pre-operative preparation and is ready-to-use as provided [11]. For this reason, Purabond and Floseal are the preferred adjuncts of use given their convenience of preparation and application. Both can also be stored easily in a refrigerator, as opposed to Tisseel, which requires storage at -20° Celsius or lower. 

Standard post-operative care involves provision of regular analgesia and encouragement of oral intake. Nasogastric tubes are removed at day 1 post-operatively if there is adequate oral intake and pain control. Patients at high risk of thromboembolism receive prophylactic dose low-molecular weight heparin post-operatively, unless otherwise contraindicated.

Statistical analysis

Continuous variables were analysed with mean and standard deviation using Microsoft Excel, and categorical variables were reported as frequencies and percentages. Due to the small sample size involved in this study, inferential statistical tests such as chi-squared or regression analysis were not performed. Descriptive outcome data analysis was performed and contextualised against existing studies in the literature. 

Literature review

An accompanying systematic review aimed to evaluate the existing literature concerning the use of topical haemostatic adjuncts in TORS. Inclusion criteria for this literature review were studies published after 1980 investigating the use of haemostatic adjuncts in TORS for adults over the age of 18 years. Both benign and malignant indications for surgery were included and all study designs were considered including randomised controlled trials and prospective or retrospective cohort studies. Papers were excluded if topical haemostatic products were not employed, concurrent systemic haemostatic agents were used (such as tranexamic acid) or studies were published in languages other than English. The primary outcome measure was the rate of post-operative haemorrhage. Secondary outcome data was collected regarding the length of inpatient stay, post-operative pain and swallowing outcomes.

**Figure 3:**
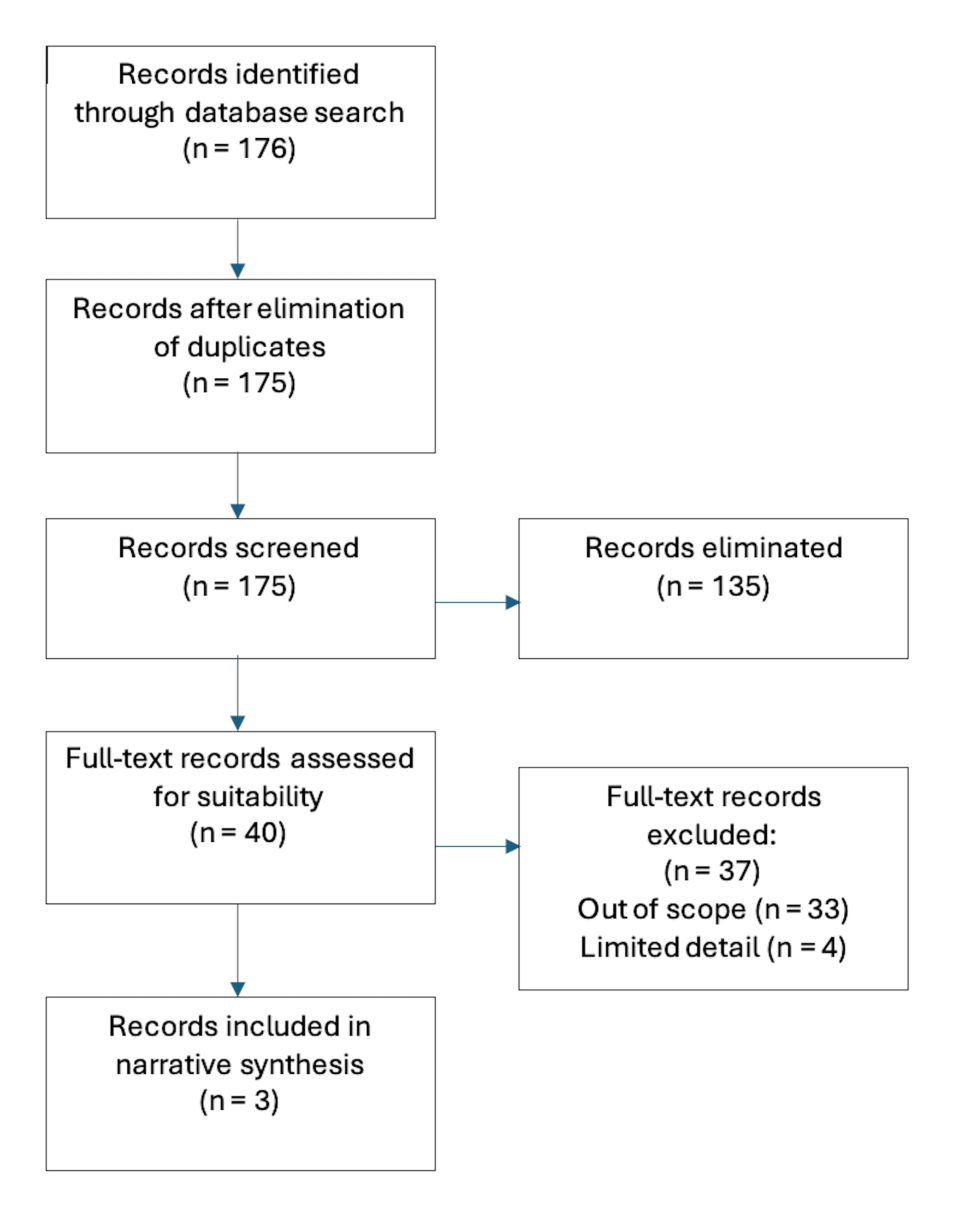
PRISMA flow diagram PRISMA: Preferred Reporting Items for Systematic Reviews and Meta-Analyses

This systematic review was conducted in accordance with guidance outlined by the PRISMA 2020 statement [18]. Two independent researchers conducted the search using the MEDLINE and OVID literature databases, and additional referenced texts were screened for inclusion. Each study meeting criteria for inclusion was assessed for risk-of-bias using the robvis web tool [19]. Boolean operators were used to perform a literature search of the above-mentioned databases. The search strategy involved the terms ("transoral robotic surgery" OR "TORS" OR "transoral surgery") AND ("haemostat*" OR "haemostasis" OR "hemo*" OR "haemo*") and this strategy was employed in both MEDLINE and OVID. Identification of 176 initial articles was completed in this way, with one article removed as a duplicate. The remaining 175 articles were screened for eligibility, leaving 40 full-text records assessed after elimination of ineligible articles. Of the 40 articles assessed, 37 were excluded as they either provided limited detail on the use of haemostatic adjuncts, or their study designs were deemed out of the intended scope of this review. Therefore, there were three final records included in narrative synthesis in this review. This is graphically depicted in a PRISMA flow diagram (Figure 3), and the included articles are listed in Table 1.

**Table 1:** List of full-text articles included in final narrative synthesis

Article name	Authors
Evaluating the role of the self-assembling topical haemostat PuraBond® in transoral robotic surgery (TORS) for oropharyngeal cancer: a case series.	Gupta et al., 2022, [20]
Topical hemostatic agents and risk of postoperative hemorrhage after transoral robotic surgery	Maza & Sharma, 2024, [21]
Transoral robotic surgery: radical tonsillectomy	Weinstein et al., 2007, [22]

## Results

Cohort study

Nineteen patients were listed for TORS procedures between October 2023 and August 2024. One of these cases was planned for a robotic approach but was instead performed via conventional approach tonsillectomy and was thus excluded, leaving 18 patients who met the inclusion criteria of our study.

The mean age of patients was 55 (SD 18.9 years). One patient died before completing the full 30-day follow-up period secondary to hospital-acquired pneumonia. TORS was most commonly indicated for suspected tonsillar primary cancer (n = 7, 38.9%), followed closely by CUP (n = 5, 27.8%) and recurrent tonsillitis (n = 5, 27.8%). One case was performed to investigate for suspected BOT cancer (5.6%). Twelve cases (66.7%) also involved another procedure alongside TORS. Most commonly, this was external carotid artery ligation (n = 8, 44.4%), panendoscopy (n = 6, 33.3%) and neck dissection (n = 4, 22.2%). Demographic and characteristics of included study participants are listed in Table 2.

**Table 2:** Demographic and characteristic data of recruited participants ECA: External carotid artery

Patient number	Age, years	Indication for surgery	Surgical procedure performed	Length of stay, days	Re-admission & reason	Haemostatic adjunct used	Estimated blood loss, ml	Complications	Notes
1	29	Recurrent tonsillitis	Robotic tonsillectomy	1	Yes – pain control	Tisseel	<100	-	
2	65	Suspected right oropharyngeal cancer	Robotic lateral oropharyngectomy & ECA ligation	3	-	Tisseel	<100	-	
3	31	Recurrent tonsillitis	Robotic tonsillectomy	1	-	Tisseel	<100	-	
4	25	Recurrent tonsillitis	Robotic tonsillectomy	1	-	Tisseel	<100		
5	70	Persistent tonsillar cyst	Robotic tonsillectomy	1	-	Tisseel	<100	-	
6	57	Suspected right oropharyngeal cancer	Robotic lateral oropharyngectomy & ECA ligation	4	-	Tisseel	<100	-	
7	34	Recurrent tonsillitis	Robotic tonsillectomy	1	-	Tisseel	<100	-	
8	59	Suspected right tonsil cancer	Robotic tonsillectomy	1	-	Tisseel	<100	-	
9	85	Suspected right base of tongue cancer	Right tongue base mucosectomy & ECA ligation	26	-	Purabond	<100	Death secondary to post-operative pneumonia	
10	56	Left neck CUP	Robotic tonsillectomy, tongue base mucosectomy, selective II-IV neck dissection & ECA ligation	6	-	Purabond	<100	-	
11	27	Recurrent tonsillitis	Robotic tonsillectomy	1	-	Purabond	<100	-	
12	64	Suspected right tonsil cancer	Robotic tonsillectomy	1	-	Purabond	<100	Haemorrhage, managed conservatively	
13	69	Right neck CUP	Robotic tonsillectomy, tongue base mucosectomy, selective II-IV neck dissection & ECA ligation	4	-	Floseal	<100	-	
14	64	Left neck CUP	Robotic tonsillectomy, tongue base mucosectomy, selective II-IV neck dissection & ECA ligation	4	-	Floseal	<100	-	
15	46	Left neck CUP	Robotic tonsillectomy, tongue base mucosectomy, selective II-IV neck dissection & ECA ligation	4	-	Floseal	<100	-	
16	61	Suspected left tonsil cancer	Robotic tonsillectomy, selective II-IV neck dissection, ECA ligation	4	-	Floseal	<100	-	
17	63	Suspected right tonsil cancer	Robotic tonsillectomy	4	-	Floseal	<100	-	
18	85	Left neck CUP	Robotic tongue base mucosectomy	4	-	Floseal	<100	-	Previous history of tonsillectomy

One patient (5.6%) presented with secondary post-operative haemorrhage which was managed conservatively. The retrospective case review identified Purabond as the haemostatic adjunct used in this case. One additional patient required readmission within 30 days due to pain (5.56%) and Tisseel was identified as the adjunct used in this case. A variety of haemostatic adjuncts were used: Tisseel (n = 8, 44.4%), Purabond (n = 6, 33.3%) and Floseal (n = 4, 22.2%). The mean length of stay was 3.7 days (SD 5.8 days). All patients resumed oral intake on day 1 post-operatively. The outlier of 26 days was secondary to a prolonged admission for hospital-acquired pneumonia, which the patient succumbed to. A summary of the outcome measures recorded is depicted in Table 3. 

**Table 3:** Outcome measures

Outcome measure	Frequency
Rate of post-operative haemorrhage	5.6% (n = 1)
Re-admission rate	11.1% (n = 2)
Mean ± SD length of stay (days)	3.7 (± 5.8)
Most frequent procedure - robotic tonsillectomy	55.6% (n = 10)

Literature review

A comprehensive search strategy identified 176 records (Figure 1). Three studies met the criteria for inclusion within this literature review. Risk of bias assessment classified all included studies as low risk. At the time of writing, the studies reviewed were published from a period spanning between May 2001 and October 2024.

Gupta et al. [20] conducted a retrospective cohort study investigating the use of a single topical haemostatic product (Purabond) in patients undergoing TORS for OPSCC. Whilst no post-operative haemorrhage or readmissions were identified following TORS, the sample size was small at just 12 patients. The average length of stay was 2.87 days (SD 0.93), which is comparable with findings in this study and those of the reported literature. All 12 patients resumed an oral diet on the first day post-operatively. 

In the US, Maza and Sharma retrospectively reviewed 80 patients undergoing TORS for both benign and oncological indications during a five-year time frame [21]. This study compared their historical use of bovine gelatin matrix with thrombin (bGMT, Floseal) against the introduction of a new porcine gelatin matrix with thrombin (pGMT, Surgiflo). There were no control patients who received standard haemostatic techniques alone. Post-operative haemorrhage rates were 7.5% (n = 6), with two cases classified as ‘severe’ (2.5%). There was a statistically significant reduction in the incidence of haemorrhagic events following the introduction of pGMT (p = 0.0183), although the difference between rates of major or severe haemorrhage between the bGMT and pGMT groups was not significant (p = 0.1196). 

## Discussion

Our experience of using haemostatic adjuncts in TORS is that they are convenient to use as an added tool to reduce the risk of peri-operative haemorrhage. Our experience found that all adjuncts assessed in this study were easy and surgeon-friendly to apply to the area in question. Use of a long-tipped applicator is helpful in accessing areas deep or lateral in the oropharynx to apply the adjunct. Another useful function of this is to enable application of adjuncts to conform to irregular wound edges, which are common in the oropharynx. 

Purabond utilises the RADA16 family of proteins to encourage haemostasis. One animal study suggested Purabond facilitated tissue healing and re-epithelialisation when applied following oral surgery [23]. This posits a question as to the potential role of haemostatic adjuncts to cause a secondary benefit of improved wound healing, an important consideration in the often-painful oropharyngeal wounds created in TORS.

Tisseel is available in a range of volumes, with smaller volumes understandably quicker to thaw. Using 4ml of Tisseel sealant is usually sufficient to coat the area of surgical resection, with enough remaining to allow repeated application if necessary. We found that one helpful caveat when using Purabond is that the application can be repeated, if necessary, directly onto an area previously treated with Purabond to encourage haemostasis. This was also observed to encourage haemostasis without the need for excessive cautery in a number of cases. We would recommend routinely applying a haemostatic adjunct in TORS, especially given the highly vascular nature of the oropharynx. 

One drawback of Tisseel is the need for frozen storage conditions, whilst Purabond and Floseal can be maintained at normal refrigerator conditions. The thawing process invariably delays the deployment of Tisseel, although anticipating the Tisseel’s use prior to thawing ensures there is no delay in its availability.

Our study is also the first to assess the role of Tisseel, a gelatin-free product, in TORS. This is important as many products utilise gelatin-based matrices, which may not be acceptable to patients on religious or dietary grounds [24]. Evidence on the efficacy of non-gelatin-based products, like Tisseel, would help support patient autonomy, enable individualised decision making and offer a wider choice to both surgeons and patients. 

Our study is limited by a small sample size and lack of a control group. Consequently, the findings of this study are difficult to extrapolate as a whole. Lack of a comparator group or standardised method of selection of adjunct use in this study means that inferential statistical conclusions cannot be reliably drawn. However, this study provides preliminary data and real-world experience on the utility of topical haemostatic agents in TORS, which is likely to represent a growing surgical field in the coming years. Formally blinded, randomised controlled trials are required to investigate the efficacy of topical haemostatic adjuncts before any evidence-based recommendations can be given on their use. Importantly, this study provides a springboard from which formally randomised studies can be conducted to assess the efficacy of these adjuncts in TORS. As TORS continues to grow in popularity, further research is required to explore whether topical haemostats are associated with improved rates of post-operative haemorrhage, faster resumption of normal oral diet, reduced post-operative analgesia and reduced length of stay. This study provides preliminary data that may be useful in guiding further research in this area. Formal, prospective studies assessing utility, cost-effectiveness and morbidity profile of the use of these adjuncts in TORS would be welcomed to glean further insight into their applicability in this use case.

Contextualising our findings in the wider literature is again challenging given the paucity of studies in this area and highlights the need for further research. The existing studies described in the literature review report similar rates of haemorrhage when compared with standard haemostatic techniques alone. However, a study by Gupta et al. on Purabond did not use a randomised format or a comparator arm and is thus limited similarly to our study [20]. Maza & Sharma did demonstrate a statistically significant reduction in the overall number of haemorrhage events when using pGMT versus bGMT [21], and this may indicate a possible fruitful area of further exploration. It is clear that the present lack of robust studies in this area means that our study highlights the need for dedicated investment and research into the use of haemostatic adjuncts in TORS.

## Conclusions

Our study is the first to assess the use of Tisseel in the context of TORS, establishing a baseline for future research. Our preliminary observational findings highlight the need for further research in this developing surgical field. As TORS becomes more established and widely undertaken, information regarding the optimal use of haemostatic adjuncts is important in devising evidence-based surgical protocols. Our study is limited in that it has a small sample size and lacks a control group. Thus, inferential statistics cannot be reliably attempted. Further prospective studies and cost-benefit analyses are required to better define the role of topical haemostatic adjuncts in the rapidly evolving field of robotic head and neck surgery.
